# Supplementary material for: MetaRibo-Seq measures translation in microbiomes
Source: Nat Commun. 2020 Jun 29;11:3268. doi: 10.1038/s41467-020-17081-z (PMC7324362; doi:10.1038/s41467-020-17081-z)
Supplement: Supplementary file 10 — Supplementary Data 7 [file 41467_2020_17081_MOESM10_ESM.zip › File2/Confidence_VeryHigh_Taxonomy/261269_out.krona.html]

Javascript must be enabled to view this page.

members
magnitude
magnitudeUnassigned
count
unassigned
taxon
rank

261269\_out

9

superkingdom
2759
9

kingdom
4751
9

9
451864
subkingdom

4
5204
phylum


SRS051031\_contig\_number\_contig-100\_21881.109140
subphylum
5302
4
1

3
155619
class

subclass
452333
2

order
2
68889

suborder
2
227334

family
2
389951

genus
80744
2

85982
2
species

341189
2

SRS013687\_contig\_number\_contig-100\_39590.84533SRS076929\_contig\_number\_contig-100\_2058.123133
varietas

36064
1
order

5250
1
family

genus
1322061
1

1
456999
species

SRS098571\_contig\_number\_49878

5
4890
phylum

4
147538
subphylum

class
4
147550

subclass
222545
1

1
37989
order

37990
1
family

1
37991
genus

species

SRS015190\_contig\_number\_6117
1
363999

subclass
222543
3

5592
3
order

3
1028423
family

genus
3
60496

3
1346820
species

SRS023914\_contig\_number\_24438SRS053356\_contig\_number\_28464SRS1041145\_contig\_number\_31196

subphylum
147537
1

1
4891
class

1
4892
order

family
1
4893

genus
1
300275

1
4955
species

SRS019685\_contig\_number\_contig-100\_4649.164487
